# Relative Reproduction Number of SARS-CoV-2 Omicron (B.1.1.529) Compared with Delta Variant in South Africa

**DOI:** 10.3390/jcm11010030

**Published:** 2021-12-23

**Authors:** Hiroshi Nishiura, Kimihito Ito, Asami Anzai, Tetsuro Kobayashi, Chayada Piantham, Alfonso J. Rodríguez-Morales

**Affiliations:** 1 Kyoto University School of Public Health, Yoshidakonoecho, Sakyoku, Kyoto City 6068501, Japan; anzai.asami.43c@st.kyoto-u.ac.jp (A.A.); kobayashi.tetsuro.6c@kyoto-u.ac.jp (T.K.); 2 International Institute for Zoonosis Control, Hokkaido University, Sapporo 0010020, Japan; itok@czc.hokudai.ac.jp (K.I.); chayada@czc.hokudai.ac.jp (C.P.); 3 Grupo de Investigación Biomedicina, Faculty of Medicine, Fundación Universitaria Autónoma de las Américas, Pereira 660003, Risaralda, Colombia; arodriguezm@utp.edu.co; 4 Clinical Epidemiology and Biostatistics, Universidad Científica del Sur, Lima 4861, Peru; 5 School of Medicine, Universidad Privada Franz Tamayo (UNIFRANZ), Cochabamba 4780, Bolivia

## 1. Introduction

The world identified the rapidly increasing incidence of the causative variant of SARS-CoV-2 Pangolin B.1.1.529 in the Gauteng province, South Africa. With as many as thirty-two notable mutations in spike protein in late November, Omicron subsequently and swiftly replaced the circulating Delta and other variants [1]. The recognition of a variant is considered to have taken place two months later than its emergence. The remarkable global spread was notable, involving 50 countries with genome surveillance capacity as of 8 December 2021.

Before replacing the Delta variant, the epidemic in South Africa was steadily downward. Considering that vaccination coverage was smaller than 30%, it was likely that a substantial fraction of the remainder of the population acquired infection naturally. Why was Omicron successful in causing a new epidemic? Understanding the transmissibility sheds light on the mechanism behind this observation. Here, we present our modelling result from an analysis of genome surveillance data in Gauteng province, South Africa, using an existing estimation technique [2].

## 2. Modelling Replacement

Genome surveillance data of Gauteng province as registered to Global Initiative on Sharing Avian Influenza Data (GISAID) was downloaded as of the end of November 2021 (Supplementary Table S1). We assumed that the effective reproduction number of the Omicron variant, *R*_omicron_(*t*) was given by multiplying a constant factor *k* to that of Delta variant, *R*_delta_(*t*), i.e., We assumed a relationship *R*_omicron_(*t*) = *kR*_delta_(*t*). As we have studied in the past [2], the fraction of genome surveillance results showing what Omicron was responsible for at a given calendar time *t*, *q*_v_(*t*) was modelled as

$$q_{v}\left(t\right)=\frac{k\int_{0}^{\infty }q_{v}\left(t-s\right)g\left(s\right)ds}{k\int_{0}^{\infty }q_{v}\left(t-s\right)g\left(s\right)ds+\int_{0}^{\infty }q_{1}\left(t-s\right)g\left(s\right)ds+k_{2}\int_{0}^{\infty }q_{2}\left(t-s\right)g\left(s\right)ds}$$

where *q*_1_(*t*) and *q*_2_(*t*) are fractions of Delta and other variants at calendar time *t*, *g*(*s*) is the probability density function of the generation time (assumed as independent of the variant with a mean of 4.7 days [3]), and *k*_2_ is the relative effective reproduction number of other variants compared with the Delta variant. Multinomial distribution was employed for maximum likelihood estimation of unknown parameters.

**Figure 1:**
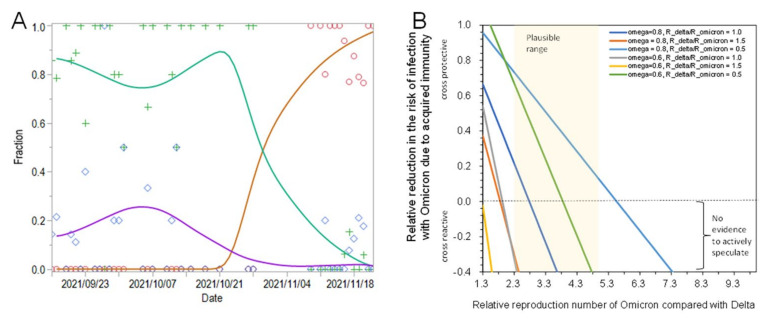
Transmissibility assessment of Omicron variant. (**A**) Estimated temporal changes in SARS-CoV-2 variant relative frequencies of Delta (Green), Omicron (Red), and other variants (Purple) circulating in Gauteng, South Africa, 16 September to 22 November 2021. Marks represent observed data, while lines are from predicted model. (**B**) The relationship between the protective effect of acquired immunity and the relative transmissibility of Omicron variant compared with Delta variant. *ω* represents the immune fraction in South Africa. *R*_delta_/*R*_omicron_ is the ratio of the basic reproduction number of Delta variant to that of Omicron, i.e., 1.5 indicating that Delta is more transmissible in a naïve population, 1.0 equally transmissible, and 0.5 indicating that the intrinsic transmissibility of Omicron is twice as large as that of Delta variant.

Figure 1A shows the comparison between predicted and observed fractions of Omicron, Delta, and other variants for 16 September–30 November 2021. *R*_omicron_(*t*) of Omicron was estimated to be 4.2 times (95% confidence interval (CI): 2.1, 9.1) greater than that of the Delta variant. The effective reproduction number of other variants was estimated as 1.3 times (95% CI: 0.7, 2.0) times greater. Alternatively, estimating the relative exponential growth rate from 18 October–30 November 2021, the Omicron variant was 3.3 times (95% CI: 2.0, 7.8) more transmissible than the Delta variant.

## 3. Transmissibility or Escape from Immune Response

What does that mean? Figure 1B illustrates how the transmission advantages of Omicron were achieved. Let *ω* be the fraction immune at the beginning of the ongoing epidemic in South Africa. $\epsilon_{delta}\left(t\right)$ is the time-dependent relative risk reduction due to acquired immunity against the Delta variant. We have

$$R_{delta}\left(t\right)=\left(1-\epsilon_{delta}\left(t\right)\omega \right)R_{0,delta}$$

where *R*_0, *delta*_ is the basic reproduction number of Delta variant which may be on the order of five or six during winter season. A similar argument applies to the Omicron variant. Then, the immune protection against Omicron variant, $\epsilon_{omicron}$, induced by acquired immunity in the present-day South Africa is estimated as

$$\epsilon_{omicron}=\frac{1}{\omega }\left(1-k\left(1-\epsilon_{delta}\left(t\right)\omega \right)\frac{R_{0,delta}}{R_{0,omicron}}\right)$$

Exploring the plausible range, $\epsilon_{omicron}$ is likely to be very small, e.g., in the order of 10–20%.

Namely, the transmission advantage of Omicron over Delta is likely gained by the mechanism of Omicron to escape from existing immunity in the population. A reduced level of neutralization against the Omicron variant among previously vaccinated individuals has been reported [4], and moreover, an increased frequency of reinfections has been demonstrated [1]. Of course, our exercise does not refute the actual elevation of the transmissibility of Omicron compared with the Delta variant. However, at least the order elevation of four times entirely due to increased transmissibility is unlikely.

## 4. Remaining Key Questions

As demonstrated here, Omicron has a substantial transmission potential to penetrate the existing herd protection due to mass vaccination in many countries and regions [5]. Key questions to be yet answered include (1) the vaccine effectiveness against Omicron, especially among countries that used different types of vaccine (e.g., messenger RNA vaccine) and (2) the clinical severity of infection across a broad spectrum of age and underlying health conditions.

## Data Availability

Not applicable.

## Supplementary Materials

The following are available online at https://www.mdpi.com/article/10.3390/jcm11010030/s1, Table S1: Submitting laboratory of GISAID data.
